# Announcement: NAIMI Nucleic Acids and Their Interactions With Metal Ions

**DOI:** 10.1155/MBD.1998.259a

**Published:** 1998


					Alghero September 5-7 1998
Scientific Program
The mini symposium is intended for researchers in the Chemistry of Nucleic Acids and Metal Ions field. The
main objective of the symposium is to bring together scientists interested in the different aspects of the
influence of metal ions on structure and metabolism of DNA. The main topics will be DNA Polymers,
nucleic metal proteins, zinc fingers and PNA.
The following distinguished speakers have already agreed to deliver a lecture:
Prof. James Allan Cowan (COLUMBUS, USA),
Metals and Nucleic Acids. New Perspectives on Nuclease Chemistry and Drug Design
Prof. Rob Kaptein (UTRECHT, THE NETHERLANDS),
NMR studies ofnucleic acid-protein interactions
Prof. Bernard Meunier (CNRS, FRANCE),
Recent advances on the mechanism ofDNA cleavage by metalloporphyrin-vector
conjugates
Prof. Thomas W. Myers (ROCHE, USA),
Manganese Activated High Temperature Reverse Transcription and PCR Amplification
Prof. Huguette Pelletier (HOUSTON, USA),
Manganese(II) and the Fidelity ofNucleic Acid Polymerases
.Prof. Bibudhendra Sarkar (TORONTO, CANADA),
Structure, function and the stability ofthe zincfinger in the retinoid receptors
In addition there will be a poster session and a number of selected oral communications. All those who wish
to participate are invited to submit an abstract to the scientific committee. The authors will be sent
notification of acceptance of posters and if they have been selected for oral presentation by 15th June 1998.
The abstract must be typed in A4 format, in a single page, centred, with 2-cm margins and with the name of
the speaker underlined. There is no formal deadline for registration: it is possible to register on site. It is
recommended however, that people wishing to attend the symposium notify their interest to Dr. Valeria
Maida as soon as possible.For further information" naimi@ssmain.niss.it
Organising committee"
F. Bonomi (University of Milano- Italy)
M.L. Ganadu, Chairperson (University of Sassari- Italy)
H. Kozlowski (University of Wrockaw- Poland)
C. Mealli (CNR, Florence- Italy)
A. Scozzafava (University ofFlorence Italy)
M. Taddei (University of Sassari- Italy)
259
Payment by means of Credit card
or
Banking transfer addressed to:
S.G.T.s.r.l.- Cariplo Ag.
Via Paoli 07100 Sassari Italy
c/c ll3/1 CAB 17201 ABI 6070
Accommodation
30 rooms are available on the campus. Other participants and their families will be accommodated in the
nearby Porto Conte or Capocaccia Hotels. The Hotels are excellently equipped with all necessary tourist
facilities such as swimming pools recreation facilities etc. As the congress will take place during the high
tourist season we suggest that you book as soon as possible to be sure to obtain a suitable room. There will
be also a social program for those attending the congress and those accompanying them. This program will
be available as soon as possible.
Prices:
Centro Ricerche Porto
Conte (Campus)
Hotel Capo Caccia,
Hotel Porto Conte
SINGLEROOM
full board
Lit. 130.000
Lit. 150.000
halfboard
Lit. 115.000
DOUBLEROOM
full board
Lit. 100.000
Lit. 115.000
halfboard
Lit. 80.000
Payment Modality
Payment should be sent to Nemapress Account No. 5629, Banca Nazionale del Lavoro- Alghero- ABI
1005 CAB 84890. Payment can be made also by Credit Card (VISA, MASTERCARD, EUROCARD,
CARTA SI).
The inscription fee can also be paid when registering at the congress.
Registration fee
Participants: Lit 250.000
Students: Lit 150.000
There are a limited number of grants available to cover expenses for young scientists (i.e. under 35).
For luther information please contact:
Peter H. Norton Phone
FAX
E-mail
+39 79 299640
+39 79 299640
peter.norton@flashnet.it
For information about travel and accomodation please contact:
Starviaggi Phone +39 79 281919
FAX +39 79 281920
E-mail Starviggi@mail.dex-net,corn
For scientific information, please contact:
Dr. Valeria Maida E-mail: noim @ssmain.uniss.it
URL" htto://www.uniss.it/web/ongressi/naim,i/naioaiaw.h;m
260

				

